# The New Direct Antiviral Agents and Hepatitis C in Thoracic Transplantation: Impact on Donors and Recipients

**DOI:** 10.1007/s40472-018-0192-y

**Published:** 2018-04-10

**Authors:** Robert L. Gottlieb, Shelley A. Hall

**Affiliations:** 1 Annette C. and Harold C. Simmons Transplant Institute, Baylor Scott & White Research Institute, Dallas, TX USA; 2 Center for Advanced Heart and Lung Disease, Baylor University Medical Center, 3410 Worth Street, Suite 250, Dallas, TX 75246 USA; 3 Department of Internal Medicine, Texas A&M Health Science Center, Dallas, TX USA

**Keywords:** Hepatitis C, Heart transplant, Lung transplant, Direct-acting antiviral, Organ allocation, Donor shortage

## Abstract

**Purpose of Review:**

The landscape of abdominal organ transplantation has been altered by the emergence of curative direct-acting antiviral agents for hepatitis C. Expansion of the thoracic donor pool to include the hearts and the lungs from hepatitis C-positive donors holds promise to increase available donor organs.

**Recent Findings:**

Case reports have documented separate lung and heart transplant patients who acquired, and then were cured of, donor-derived hepatitis C using these newer, more effective therapies. Single sites and national consortia are underway to help make this approach part of the standard-of-care. Pangenotypic therapies may simplify the paradigm.

**Summary:**

Organs from donors with active hepatitis C viremia are likely suitable for transplant as long as the organ is otherwise acceptable. Best-practices for “informed-risk” transplant include a team-based approach and a selection of the antiviral regimen based on insurer’s formulary, potential drug interactions, and genotype.

## Introduction

Organ offers from donors with prior or chronic hepatitis C virus (HCV) exposure have been historically underutilized for orthotopic heart transplantation and lung transplant owing to post-transplantation risks [1, 2•]. The use of HCV antibody-positive (Ab+) donors was associated with attenuated survival benefit after heart transplant and increased coronary allograft vasculopathy in the era before new highly effective direct-acting antiviral agents (DAAs) were developed [3, 4]. These DAAs target multiple steps in the HCV replication life cycle [5•]. Early data described within this review provides validation that newer, well-tolerated, oral direct-acting antivirals (DAAs) may transform thoracic transplant outcomes after donor-derived HCV transmission.

## Detection of Hepatitis C

The historical literature on hepatitis C-positive organ donors is confounded by the natural history of hepatitis C and our incremental diagnostic ability to detect and characterize hepatitis C. Past ability to only detect hepatitis C antibody (HCV Ab) indicates a history of infection, without distinguishing whether the individual has spontaneously cleared the infection, has cleared the infection with pharmacotherapy, or still has ongoing viremia. Also, there is a 4–6-week window period, during which a recently infected patient may have ongoing viremia while not yet demonstrating seropositivity.

Now that HCV nucleic-acid testing (NAT), a polymerase chain reaction (PCR)-based approach to detecting viral activity, is widely available and used on all US donor organs, transplant centers have more relevant information about the donor. An updated assessment is required to reflect current practice and therapeutic opportunities given our current pharmacologic armamentarium.

Because HCV is a non-integrating single-stranded plus-sense RNA virus, there is no viral reservoir once sustained viral remission is achieved. The current hepatology and virology literature has evolved to define a primary standard endpoint of sustained viral load less than the lower limit of detection 12 weeks following the completion of therapy (SVR-12), i.e., the results of viral testing by PCR 12 weeks off-therapy regardless of duration of therapy [6•].

## Thoracic Transplantation in HCV-Positive Recipients Pre-transplant (Before the Emergence of DAA Therapy)

The success of solid organ transplant has been assessed by whether the donor, recipient, or both had a history of hepatitis C (i.e., the direction of mismatch). From the recipient perspective, lung transplant recipients who had at least a history of hepatitis C positivity prior to transplant demonstrated poorer survival compared to hepatitis C-negative recipients following transplant during the era of 1994–1999 (median survival 1.7 vs 4.5 years; *p* = 0.004), but not the era of 2000–2011 (*p* = 0.100) [2•]. Thus, the risk/benefit ratio is changing, and current data and prospective trials will be required to facilitate an understanding of the transplant biology risks and benefits in the modern era of DAA availability.

During the period of 1991–2014, only 2% of heart transplant recipients in the USA had a history of HCV exposure pre-transplant. Retrospective analysis of this cohort (without distinction between recipients with chronic hepatitis C vs those that had either been treated or spontaneously cleared their hepatitis C) indicated that while those recipients with a history of hepatitis C at some point pre-transplant had improved survival relative to the natural course of heart-failure, the magnitude of the transplant benefit was attenuated relative to lifelong HCV negative recipients (64.3 vs 72.9% and 43.2 vs 55% at 5 and 10 years; *p* < 0.01), respectively [7•].

## Virology of Hepatitis C and DAAs

There are at least 6 HCV genotypes with more than 50 subtypes. Genotype 1 (consisting of types 1a and 1b) is the most common genotype worldwide, although there is significant geographic variability. In the USA, genotype 1 subtypes account for ~ 75% of the cases, genotypes 2 and 3 subtypes represent 20–25% of cases, and the remainder of cases are genotypes 4, 5, and 6. Like many other plus-sense viruses, the genome is divided into a 5’ portion that encodes structural proteins and a 3’ portion encoding non-structural (NS) proteins. The genome is transcribed into a polyprotein that is then cleaved by a combination of the viral NS3/4A protease and the cellular host’s endogenous peptidases, yielding the individual structural and non-structural proteins.

Oral DAAs vary in their efficacy against various viral genotypes. The ideal anti-HCV therapy post-transplant is one that covers all genotypes efficaciously while generating minimal drug interactions or contraindications. The more easily understood and managed regimens of DAAs pharmacologically target two or more different NS proteins (Table 1).

**Table 1:** Individual classes of the direct-acting antiviral (DAA) components, along with their generic names. DAAs include two or more pharmaceuticals in combination to target a viral protease (NS3/4A), a viral replication complex protein (NS5A), or the viral RNA-dependent RNA polymerase (RdRP, NS5B). Genotype specificity is not intended to be exhaustive and may reflect in vitro activity or require other adjuncts for coverage (some requiring ribavirin and/or pegylated interferon still). Although DAAs are listed independently, this does not imply that any two can be combined; permutations tested are too complex to list in a table. Numbers in parentheses denote the genotype specificity

Class of DAA	Generic drug name	Common abbreviation
NS3/4A protease inhibitors	Boceprevir (1)	BOC
	Glecaprevir (1–6)	GLE
	Paritaprevir (1, 4) with ritonavir	PTV/r
	Simeprevir (1,4)	SMV
	Telaprevir (1)	TVR
	Voxilaprevir (1–6)	VOX
	Grazoprevir (1, 3, 4)	GZR
	Asunaprevir (1, 4)	ASV
NS5A replication complex inhibitors	Ombitasvir (1, 4)	OBV
	Pibrentasvir (1–6)	PIB
	Daclatasvir (3)	DCV
	Elbasvir (1, 4)	EBR
	Ledipasvir (1)	LDV
	Velpatasvir (1–6)	VEL
NS5B RdRp nucleotide-analog inhibitors	Sofosbuvir (1–4)	SOF
NS5B RdRp non-nucleoside/non-nucleotide inhibitors	Beclabuvir (1,3–6)	BCV
	Dasabuvir (1)	DSV

Until the summer of 2016, effective treatment of HCV, even in the general population, required knowledge of viral genotype. Those treatment regimens with broader activity across genotypes required complex tailoring (e.g., addition of ribavirin, with potential drug interactions), while activity against HCV genotype 3 remained a particular challenge. More recently, pangenotypic regimens (i.e., regimens that are efficacious regardless of genotype) have become FDA-approved for chronic hepatitis C. These regimens now include sofosbuvir/velpatasvir (SOF/VEL), which is a fixed-dose combination of sofosbuvir, a nucleotide-analogue NS5B inhibitor, and velpatasvir, an NS5A replication complex inhibitor, with activity against all 6 major HCV genotypes [8•]; sofosbuvir/velpatasvir/voxilaprevir that includes an NS3/4A protease inhibitor (SOF/VEL/VOX), and the NS3/4A-inhibitor plus NS5A inhibitor combination of glecaprevir and pibrentasvir (GLE/PIB) (Table 2). The sofosbuvir-based regimens (SOF/LDV, SOF/VEL) are most common in the existing case reports, with elbasvir/grazoprevir (EBR/GZR) used for transplant of HCV NAT+ donor heart via an enrolling trial. SOF/VEL has pangenotypic coverage, as does GLE/PIB, but GLE/PIB is not recommended with cyclosporine doses > 100 mg/day. Nevertheless, due to insurance constraints, we have also deployed GLE/PIB to counter donor-derived HCV genotype 3a in a heart transplant recipient who underwent repeat heart transplantation from a best-interest standard, informed-risk HCV+ donor (Gottlieb RL, Hall SA, Trotter JF, Haley CC, and Wada SY, unpublished observation). SOF/VEL/VOX is also pangenotypic but without known use in thoracic transplant to date.

**Table 2:** Select DAA regimens, including pangenotypic regimens, and status of thoracic clinical trials

			Pangenotypic		
Tradename	Harvoni® (Gilead Sciences, USA)	Zepatier® (Merck, Inc., USA)	Epclusa® (Gilead Sciences, USA)	Vosevi® (Gilead Sciences, USA)	Mavyret® (AbbVie, USA)
FDA approval date			June 2016	July 2017*	August 2017
Genotype	1, 4–6	1,4	ALL (1–6)	ALL (1–6)	ALL (1–6)
NS3/4A protease inhibitors		GZR		VOX	GLE
NS5A replication complex inhibitors	LDV	EBR	VEL	VEL	PIB
NS5B RdRp nucleotide-analog inhibitors	SOF		SOF	SOF	
Heart (single center)		NCT03146741	NCT03086044		NCT03382847
		NCT03026023			
Heart (consortium)			NCT03383419		
Lung (single center)			NCT03112044		
			NCT03086044		
Lung (consortium)			Pending		

*Approved for re-treatment of hepatitis C

## What is in a DAA Label? Semantics and Cost

Prescription of DAA inhibitors to facilitate transplantation of organs from donors with active hepatitis C viremia was a natural extension of the experiences of our colleagues in abdominal transplant as DAAs be used with our without ribavirin in patients with significant hepatic insufficiency or cirrhosis, as dictated by their Child-Pugh score. However, the FDA label for all of the DAA products is for chronic hepatitis C, rather than acute hepatitis C. From an insurer’s perspective, these therapies have been extremely expensive ($80,000–$100,000) and are only now beginning to demonstrate pricing elasticity with an abrupt and ongoing dramatic price-drop due to competitive market influences. As a consequence, insurers have naturally had incentive to keep each dispensing on-label, and each payor has their own strict criteria for use, frequently having preferred DAA regimens based on contracting and genotype. Moreover, chronicity has been mandated as perhaps 25% of acute hepatitis C infections will be cleared spontaneously, resulting in the potential for substantial savings to the insurer by ensuring chronicity. Indeed, even healthcare workers who might contract hepatitis C by an inadvertent needle stick do not have access to an FDA-labeled DAA therapy. One can extrapolate, however, if enough cumulative safety and efficacy data arises for profoundly immunosuppressed transplant patients who may be treated before classic definitions of chronicity ensue, then perhaps by performing this research on our patients, we may in return receive benefit by the development of a label that will mitigate our work-place hazard.

## Lung and Heart Transplant in the Age of DAA: Pangenotypic Regimens Enable Paradigm Shift

The re-emergence of thoracic organ transplantation using HCV-viremic donors is a natural extension of the advancement in abdominal organ transplantation. To our knowledge, the first report of DAA deployment in thoracic transplantation involved the best-interest use of a lung allograft from a hepatitis C-positive donor transplanted into a seronegative recipient by Dr. Cypel’s group in Toronto, Canada [9•]. The HCV genotype was unknown until after transplantation, when it was determined to be genotype 1a, enabling the use of ledipasvir-sofosbuvir (LDV/SOF). Whereas HCV genotype 3 is responsible for < 10% of hepatitis C infections in the USA, it is relatively enriched in the donors becoming available as a consequence of the intravenous drug overdose epidemic, and thus our Canadian colleagues could not have guaranteed that they would have a DAA regimen to treat their patient at that time.

Although presence of HCV antibodies and viremia assessed with the use of nucleic-acid testing (NAT) are disclosed, genotype and viral titers are rarely available at the time of organ allocation given the limited time constraints. Transplanting a heart or lung laden with an unknown genotype of HCV with the intention to deploy a DAA regimen to eradicate donor-derived HCV infection after transplantation would have been a daunting prospect until the advent of simpler pangenotypic therapies.

In summer of 2016, the once-daily, oral, fixed-dose SOF/VEL with pangenotypic activity against all 6 major HCV genotypes became available commercially [8•]. Shortly thereafter, a critically ill patient elected to expand her potential donor pool to include donors regardless of hepatitis C status. Within days, she received a suitable allograft from an HCV-viremic donor (HCV Ab+, NAT+) based on the best-interest standard, without knowledge of the HCV genotype a priori and with a plan to institute a pangenotypic agent at a suitable time post-transplant. We at Baylor University Medical Center (led by Drs. Hall and Gonzalez-Stawinski) were the first to report this experience of an informed transplant of an HCV-viremic heart into an HCV-naïve donor, with subsequent SVR-12 using a DAA regimen, and further reported the viral kinetics [10•]. The availability of a well-tolerated pangenotypic regimen was instrumental in ensuring eradication of the donor-derived HCV, as our index patient contracted donor-derived HCV genotype 3, suitable only for treatment with SOF/VEL, the only pangenotypic DAA regimen available at that time. This particular donor:recipient pair represented a “triple mismatch” with CMV mismatch [donor positive, recipient negative (CMV D+/R−)], hepatitis B-positive (into a HBV vaccinated recipient), and HCV donor NAT+, recipient NAT−. That index patient has never been rehospitalized since her transplant. Interestingly, our next HCV-naive patient to undergo an informed, HCV-viremic heart transplanted according to best-interest standard also contracted donor-derived HCV genotype 3a, again confirming the imperative nature of pangenotypic regimens to ensure optimal organ allocation.

Recently, Dr. Lindenfeld and colleagues have provided data in support of our strategy. They report their case series of 11 HCV NAT-positive donors in NAT-negative recipients (one of whom had a pre-transplant history of prior HCV with SVR). In 9 of these patients, HCV viremia ensued, and excluding a patient who died of autopsy-proven pulmonary embolism (with a pre-transplant history of thrombophilia), the remaining 8 patients all achieved SVR-12 [11•]. Further illustrating the need to parse the definition of HCV-positive donors in the literature, they also described an additional two patients who received transplants from cardiac donors who were HCV Ab-positive but HCV NAT-negative. Those two recipients did not demonstrate any donor-related transmission.

Extrapolating from our hepatology colleagues, Uriel and colleagues describe treating a patient suffering with cardiomyopathy and concomitant cirrhosis and hepatocellular carcinoma as stigmata of hepatitis C genotype 1b with an ostensibly HCV-positive donor (although details are not described to indicate donor viremia or genotype). Following dual-organ transplantation of the heart and the liver, that patient underwent a course of LDV/SOF resulting in SVR-12 of at least the pre-existing genotype 1b viremia [12•].

Intriguingly, Schlendorf et al. report antibodies as early as 1-day post-transplant [11•]. We have also seen the same phenomenon in our patients who were seronegative pre-transplant (Gottlieb and Hall, unpublished observations) and agree with their interpretation that it is physiologically too soon for those antibodies to have formed in the recipient [13, 14], and agree that they likely represent donor-derived antibodies; we interpret this as indicating adsorbed antibodies sequestered within the donor heart at the time of transplant.

## CDC Increased-Risk Donors as Inadequate Terminology: a Proposal to Use “Informed Risk”

The term Centers for Disease Control (CDC) increased-risk (IR) donor (formerly labeled CDC high-risk) is an incomplete description of the communicable-disease risk of a potential donor. It is a particularly flawed term as it only applies to social history and behaviors, irrespective of known serologic or virologic data. A donor may be known to carry hepatitis C viremia (HCV NAT+) and not even be labeled a CDC IR donor. To address this gap in terminology, at our center, we have begun using the term “informed-risk” donors as, even if not labeled as an IR donor, we inform the recipients of the donor’s viremic status due to its direct consequence on their post-transplant care. We only allow these pairs to proceed after a consent conversation that addresses the anticipation that the recipient will be infected, and we will then take measures to control the viremia. With hepatitis B-positive donors, we proceed with HBV NAT+ informed-risk donors as a standard-of-care with entecavir as our typical antiviral and further mitigate risk by confirming or recommending hepatitis B vaccination early in their transplant candidacy evaluation, as their physiologic condition permits. With HCV NAT+ donors, we previously performed heart transplant from HCV informed-risk donors based on the best-interest standard, but are now enrolling patients in our multicenter prospective consortium trial, TROJAN-C, NCT03383419. This trial will offer patients the first suitable donor organ offer, irrespective of hepatitis C status, with an intention to initiate treatment with a pangenotypic regimen, once-daily sofosbuvir 400 mg/velpatasvir 100 mg (Epclusa, Gilead USA), within 14 days of detectable viremia. The trial is sponsored by Baylor Research Institute and will enroll patients at Baylor University Medical Center, Cedars Sinai, and Duke University Medical Center.

Now that pangenotypic agents are available, Cypel’s group at University Health Network Toronto General Hospital building on their prior experience and investigating the safety and efficacy of lung transplant of HCV-positive donors into HCV NAT− recipients using SOF/VEL as well (NCT03112044).

## Interaction Cautions: Amiodarone, Cyclosporine, and Compliance

To date, we are aware of thoracic transplant-associated, donor-derived hepatitis C infection in thoracic organs being treated with LDV/SOF, SOF/VEL, GLE/PIB, and EBR/GZR, each with their own concerns. Sofosbuvir may interact with amiodarone to result in bradycardia. We have designed our prospective, randomized trial for hepatitis C-positive heart transplantation to mitigate this risk, perhaps more cautiously than will be ultimately proven necessary, as transplant cardiologists are already attuned to the risk of allograft bradycardia. We speculate that the high basal resting heart-rate of a post-vagotomy transplanted heart might mitigate this risk. However, it would remain a concern for SOF-based antiviral therapies after hepatitis C-positive lung transplantation, as many lung transplant patients develop postoperative paroxysmal atrial fibrillation that requires heart-rate control with beta-blockers or calcium-channel blockers. Thus, the bradycardia concern may be more problematic in lung transplant. The pangenotypic GLE/PIB regimen is notable for interactions with > 100 mg cyclosporine daily dose, as cyclosporine inhibits the P-glycoprotein mediated clearance of GLE/PIB, although a thoracic trial has begun (see Table 2). Compliance is necessary to mitigate the risk of DAA-mediated selection of resistant variants.

## Viral Cautions: HBV Re-activation, Fibrosing Cholestatic Hepatitis

A critical concern involves patients previously infected with hepatitis B virus (HBV). DAA therapy is well known to potentiate the risk of hepatitis B re-activation, a marked clinical concern with overt HBV surface antigen positivity (HBV SAg+) occurring in 12% of HBV SAg+ patients treated with DAA, with greater risk after transplant immunosuppression [15•]. Each center should have its plan in place to either prophylactically suppress HBV re-activation with antivirals such as entecavir during the period of treatment, or at least survey periodically with quantitative HBV PCR to ensure that any re-activation is addressed in a timely fashion [16•]. At the current juncture, centers might consider excluding candidates for non-hepatic solid organ transplant with detectable HBV viremia (defined as either HBV SAg+ or HBV PCR+) from informed-HCV NAT+ transplant barring exceptional circumstances, until further data is available.

Independent of hepatitis B status, care should also be taken to monitor hepatic function tests to enable early recognition of acute fibrosing cholestatic hepatitis, an as yet unquantified risk of transplant-derived acute viral hepatitis C infection in thoracic transplantation, but occurring in approximately 10% of liver transplant recipients who are HCV NAT+ at the time of transplant (reviewed in Hori et al. [17•]). There is hope that DAAs will mitigate the risk.

## Ethics of Utilizing Hepatitis C-Positive Donors; Societal, Donor, and Recipient-Specific Aspects

Utilization of organs from hepatitis C-positive donors requires due attention to multiple domains of the ethical frameworks of social justice, beneficence, non-maleficence, and respect for persons. From a social justice perspective, thoracic organs for transplantation remain a critically scarce resource. In the USA, this resource is managed through the United Network for Organ Sharing (UNOS). Increasing the chances that any particular organ is accepted for transplant serves the societal interest of decreasing this shortage. But is the general public as a whole then placed at risk? Does transplantation into a host that will be iatrogenically immunocompromised result in a human reservoir teeming with hepatitis C viremia that would risk the general community? Fortunately, the general public is unlikely to be endangered. First, the mechanism of transmission of hepatitis C requires blood-borne transmission. Household contacts are at low risk for transmission as long as they observe standard universal precautions by avoiding unsafe practices such as sharing razors and ensuring safe disposal of needles and sharps, along with the use of barrier methods such as gloves in case of exposure to bodily fluids and oozing or bleeding postoperative wounds (such as chest tubes and sternotomy wounds).

Transplant recipients are generally amply vetted to ensure a much higher degree of compliance with pharmacologic regimens than the unselected general public and by surveillance of immunosuppressant drug levels, are assessed routinely for global pharmacologic compliance, thereby increasing the percentage of therapeutic time-under-the-curve. While RNA viruses demonstrate replication errors and run the risk of developing resistance-mutations, there is a plethora of new DAAs that can be used to tailor therapy in the event that a resistance mutation to a particular therapy emerges. Furthermore, the sheer number of persons harboring hepatitis C and the total viremic load contained within the general public dwarfs the total number of virions that could develop in the post-transplant population. If multi-drug resistance develops, it is statistically far more likely to emerge from the general public, rather than recipients of donor-derived hepatitis C infection.

## Non-maleficence and Informed Consent

We have taken great pains in designing our multicenter consortium trial, TROJAN-C (NCT03383419 “Transplant of Redeemed Organs by Judicious Administration of New Direct-Acting Antivirals for Hepatitis-C Heart Recipients”) to ensure patients are offered a clear and informed choice as to whether to enroll in a trial of the first available suitable organ, regardless of hepatitis C status. Although tempting to start therapy as soon as the recipient is liberated from the ventilator, we have been concerned that very early initiation of antiviral therapy may contribute to viral resistance if postoperative nausea or gastrointestinal complications limit the ability of the patient to tolerate uninterrupted oral therapy. We also do not wish to commit patients to an extended course of therapy if it is possible that they may not have contracted the virus. Therefore, we have designed our trial to initiate therapy within 14 days of quantifiable viremia, which is still far sooner than deemed necessary by our hepatology colleagues. It is interesting to compare HCV transmission to that of high-risk CMV donor+, recipient− pairs. In those cases, we know that CMV is indisputably associated with decreased cardiac allograft survival, with risk of coronary allograft vasculopathy (CAV), and yet the best we can hope for following transmission is immunity and control of re-activation of a somatically-integrated reservoir, whereas with HCV, we can offer the prospect of true cure.

## Did Immunologic or Donor-Factors Present at Transplant Attenuate Benefit from HCV-Positive Donors in the Pre-DAA Era?

Existing literature suggests that mycophenolate may have increased the risk of long-term adverse cardiac transplant outcomes after donor-derived HCV transmitted at the time of cardiac transplantation in the pre-DAA era (reviewed in [1]). Preliminarily, it appears unlikely that mycophenolate complicates outcomes after donor-derived HCV treatment with the use of DAAs. While we utilized azathioprine rather than mycophenolate as an antimetabolite in our first experience because of a high-risk CMV mismatch [10•], the Vanderbilt group was successful in achieving SVR despite concomitant mycophenolate [11•]. An additional confounder may be that donors infected with hepatitis C, vis-à-vis their risk factor for having contracted HCV, may also have a risk factor for accelerated but occult atherosclerosis. At our center, we perform baseline IVUS at 6-week post-heart transplantation so that we can distinguish donor-derived abnormalities from the time of transplant from those acquired as an immunologic consequence in the interval from the baseline study to 1 year. Although our TROJAN-C clinical trial does not mandate intravascular ultrasound (IVUS) of the cardiac allograft coronary arteries if not performed as standard-of-care, those centers in our consortium that do perform IVUS will enable us to hopefully better distinguish between these two possibilities.

## Future Directions

An unaddressed question is whether anything can be done, upstream of transplant, or peri-operatively in donor and recipient, to minimize the risk of hepatitis C transmission in HCV NAT-positive donors. Once brain death is declared in the donor, the ongoing cost of care to sustain the organs of the decedent in suitable condition for thoracic transplantation is typically borne by the organ procurement agency, and costs of pharmacotherapy would likely be prohibitive at current costs. If cost was not an issue, then an intriguing, but untested, question is whether prior to organ procurement, the donor might be pretreated with DAAs, potentially even at higher-than-typical doses, to minimize the chance of donor-derived infection of the non-reservoir organs (i.e., non-hepatic). The duration of such a potential pre-treatment is necessarily limited by the time-sensitive nature of the organ procurement process. It is unlikely that time would permit adequate and full viral suppression in the donor. While organs are frequently placed on ice, there are also ex vivo perfusion strategies for both cardiac and pulmonary allografts, as well as renal allografts. Perhaps adjunctive post-procurement therapies such as addition of DAAs into the perfusate of ex vivo organ preservation systems could further decrement infectiousness. A further limitation to this approach, however, is that even if the recipient were to be pretreated with the antiviral agent at the time of organ offer acceptance, the recipient will necessarily become NPO and without consistent and reliable access to GI absorption of the antiviral agent at the time of transplant and immediate postoperatively. If such sequential donor, ex vivo, and recipient therapies were effective, however, they could promise to be cost-effective by decreasing the total number of doses required to achieve SVR and/or block infection. These questions are necessarily speculative at this point and would require prospective research. Dr. Cypel’s group appears to be addressing a preliminary to this question via normothermic ex vivo lung perfusion of lungs from HCV+ donors, but HCV is highly infectious with direct blood-blood or transplant organ-to-blood contact, and their strategy appears to be merely dilutional, without adjunctive DAA in the perfusate (NCT03112044).

## Conclusion

The transplant community, including the thoracic transplant circle of care, is undergoing a seismic shift in standard practice. Hepatitis C donor or recipient viremia, until recently a marked risk (and center-specific contraindication) for thoracic transplant, is now promising to facilitate transplant, minimize the donor shortage, and decrease recipient wait times. If multicenter consortium trials such as TROJAN-C confirm the safety and efficacy of using DAAs to cure donor-derived hepatitis C in thoracic organ recipients, a new standard-of-care may emerge. Once all centers are able to offer this therapy safely and economically, then more donor offers can be utilized, fulfilling the social justice need for stewardship of a scarce resource. In the interim, HCV may be viewed as a “helper virus” enabling shorter wait times at those centers offering informed transplant of HCV-viremic organs on a research basis, and for the most critically ill, may be utilized on a best-interest standard with the promise, albeit not guarantee, of post-transplant treatment to cure defined as SVR-12, without reservoir, in contrast to CMV. To mitigate risk, thoughtful deliberation and conversations with the recipient and family are recommended, with appropriate monitoring for viral and pharmacologic interactions and physiologic side-effects in a multidisciplinary team approach including transplant pharmacists. Open collaboration between centers, facilitated by transplant societies, is bringing post-modern advances in transplant hepatology and infectious disease to bear fruit in thoracic transplant.
